# The protein kinase Mζ network as a bistable switch to store neuronal memory

**DOI:** 10.1186/1752-0509-4-181

**Published:** 2010-12-31

**Authors:** Hideaki Ogasawara, Mitsuo Kawato

**Affiliations:** 1 National Institute of Information and Communications Technology, 2-2-2, Hikaridai, Seika, Kyoto 619-0288, Japan; 2 ATR Brain Information Communication Research Laboratory Group, 2-2-2, Hikaridai, Seika, Kyoto 619-0288, Japan; 3 Current Address: Pharmacovigilance Department, Astellas Pharma Inc., 3-17-1, Hasune, Itabashi, Tokyo 174-8612, Japan

## Abstract

**Background:**

Protein kinase Mζ (PKMζ), the brain-specific, atypical protein kinase C isoform, plays a key role in long-term maintenance of memory. This molecule is essential for long-term potentiation of the neuron and various modalities of learning such as spatial memory and fear conditioning. It is unknown, however, how PKMζ stores information for long periods of time despite molecular turnover.

**Results:**

We hypothesized that PKMζ forms a bistable switch because it appears to constitute a positive feedback loop (PKMζ induces its local synthesis) part of which is ultrasensitive (PKMζ stimulates its synthesis through dual pathways). To examine this hypothesis, we modeled the biochemical network of PKMζ with realistic kinetic parameters. Bifurcation analyses of the model showed that the system maintains either the up state or the down state according to previous inputs. Furthermore, the model was able to reproduce a variety of previous experimental results regarding synaptic plasticity and learning, which suggested that it captures the essential mechanism for neuronal memory. We proposed *in vitro *and *in vivo *experiments that would critically examine the validity of the model and illuminate the pivotal role of PKMζ in synaptic plasticity and learning.

**Conclusions:**

This study revealed bistability of the PKMζ network and supported its pivotal role in long-term storage of memory.

## Background

Protein kinase Mζ (PKMζ) is increasingly drawing attention as a molecule that maintains neuronal memory for an extremely long period of time [1]. It is a brain-specific atypical protein kinase C (PKC) isoform that lacks a regulatory domain, rendering it constitutively active [2]. PKMζ enhances excitatory postsynaptic currents (EPSCs) and leads to the long-term potentiation (LTP) of synapses by stabilizing α-amino-3-hydroxy-5-methyl-4-isoxazolepropionic acid (AMPA)-type glutamate receptors through an N-ethylmaleimide-sensitive factor (NSF)/GluR2-dependent pathway [3-5]. The messenger ribonucleic acid (mRNA) for PKMζ is found in various brain areas, including the hippocampus, striatum, neocortex, thalamic nuclei, and cerebellar cortex and localizes to spiny dendrites of neurons [6]. PKMζ is translated within only ten minutes in response to LTP-inducing stimuli [2,7], suggesting its local synthesis.

The use of a specific inhibitor, ζ-inhibitory protein (ZIP) [8], has elucidated the pivotal role of PKMζ in synaptic plasticity, learning, and memory. The late phase of LTP (L-LTP) in a hippocampal slice is reversed by ZIP administration [9], indicating that LTP maintenance requires PKMζ. PKMζ plays crucial roles in various modalities of learning, including spatial memory of the hippocampus and fear conditioning of the basal lateral amygdala, as evidenced by memory erasure following ZIP microinjection [10]. In rats, consolidated memory is sensitive to ZIP for at least one month [11].

PKMζ appears to constitute a positive feedback loop [1,12,13]. ZIP administration prevents hippocampal neurons from expressing PKMζ protein when these neurons are treated with a tetanus that would normally induce LTP and PKMζ expression [12], indicating that PKMζ activity is necessary for PKMζ synthesis. We previously posed the possibility that the PKMζ network is bistable [13], since biochemical positive feedback loops often offer bistability [14,15]. Bistable positive feedback loops of enzymatic reactions may provide a basis for cellular memory [16,17].

Our previous model [13,18] conceptually illustrated that memory plasticity and stability can be both achieved by a cascade of multiple nonlinear or bistable dynamics that have various time constants and are connected in tandem in the order of fast to slow. Once the cascade is stimulated, activity is transmitted from a fast dynamic to a slower dynamic before the faster dynamic loses its activity; finally, the slowest dynamic is turned on. Hippocampal LTP appears to occur in line with this model. LTP-inducing stimuli trigger a supralinear calcium increase in dendritic spines that lasts for seconds [19-22]. Then, calcium activates protein kinases such as Ca^2+^/calmodulin-dependent protein kinase (CaMKII) in a supralinear manner and maintains their activity for tens of minutes [23,24]. Finally, CaMKII and other protein kinases induce longer-lasting PKMζ expression [12] probably in an all-or-none manner [13].

To evaluate our hypothesis that the PKMζ network is bistable and functions as neuronal memory, we performed simulations and bifurcation analyses in the Results section. Very simple though our model was, it was able to reproduce various experimental results. Moreover, in the Discussion section, we proposed yet to be done experiments that would critically examine our hypothesis. Although ZIP is regarded as a specific inhibitor of PKMζ, it might inhibit other protein kinases as well. In this paper, therefore, we use the term 'PKMζ' to collectively refer to ZIP-sensitive protein kinases including PKMζ.

## Results

### Description of the model

**Figure 1:**
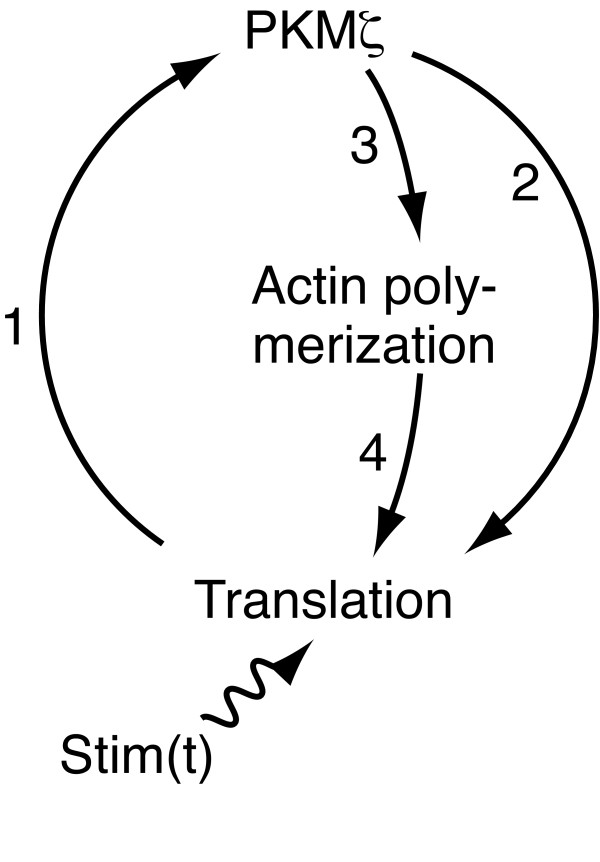
**The PKMζ network model**. See text for explanation.

Figure 1 illustrates the molecular pathways of the PKMζ network model. The model is described by three ordinary differential equations (ODEs), Equations 1-3 (see Methods). A time-dependent variable, Stim(t), represents the aggregate activity of protein kinases that trigger PKMζ expression, such as CaMKII, PKC, and mitogen-activated protein kinase (MAPK) (Figure 1, arrow 1) [2,7]. In reality, these protein kinases act through various pathways to turn on the PKMζ network. However, since the purpose of the model was to mathematically analyze the dynamics of the network, kinasic activation of the network was simplified as a single variable. Experiments have shown that the PKMζ protein stimulates translation of its own mRNA into protein (Figure 1, arrow 2) [25], forming a positive feedback loop. At the same time, its PKC activity presumably promotes actin polymerization (Figure 1, arrow 3) [26-29], and F-actin (actin polymer) facilitates general protein synthesis (Figure 1, arrow 4) [30,31]. This convergence of the two pathways is likely to make the system more sensitive to differences in stimulus size than the standard Michaelis-Menten kinetics (ultrasensitivity). A mathematical theory states that a combination of ultrasensitivity and positive feedback possibly results in bistability [15], which we examine in the following section.

### Bistability of the PKMζ network

**Figure 2:**
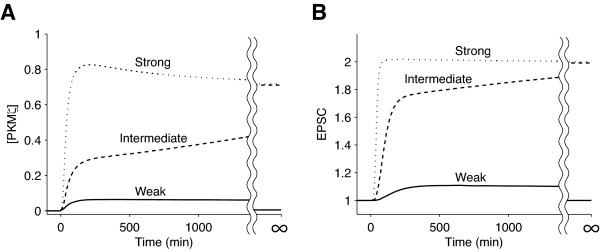
**Time courses of A) [PKMζ] and B) EPSC amplitude**. A weak (Stim = 5, solid line), intermediate (Stim = 25, dashed line) or strong (Stim = 125, dotted line) stimulus was given for 30 minutes. Stimulus strength, [PKMζ], and EPSC amplitude are unitless values.

We stimulated the model with a square wave, Stim(t) = 5, 25 or 125 (0 ≤ t < 30), to observe the time course of [PKMζ] (Figure 2A). EPSC amplitudes (Figure 2B) were estimated by time-integrating [PKMζ] (Equation 4 in Methods). In this model, the concentrations of molecules, strength of Stim, and EPSC are unitless values, and EPSC amplitudes of 1 and 2 correspond to the unpotentiated and potentiated states, respectively. When the stimulus was weak (Stim = 5), [PKMζ] rose transiently but ended up at zero, and EPSC was enhanced transiently. When the stimulus was intermediate (Stim = 25), [PKMζ] reached a value of 0.72 asymptotically, and EPSC amplitude reached a value of 2. When the stimulus was strong (Stim = 125), [PKMζ] overshot before asymptotically reaching a value of 0.72, and EPSC amplitude approached a value of 2 asymptotically.

**Figure 3:**
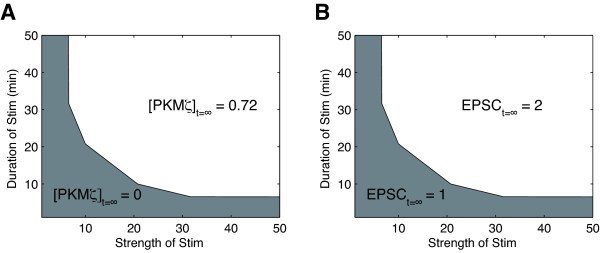
**Steady-state A) [PKMζ] and B) EPSC amplitude after various strengths and durations of Stim were given**. The strength and duration of Stim are shown on the abscissa and the ordinate, respectively.

We then varied the duration and strength of Stim(t) to see whether [PKMζ] can reach any steady state other than [PKMζ]_t = ∞ _= 0 or 0.72. In the two-dimensional parameter space (Figure 3A) with respect to the strength (abscissa) and duration (ordinate), [PKMζ]_t = ∞ _was either 0 (gray) or 0.72 (white), and not intermediate anywhere. Stimuli that were longer or shorter in duration required a weaker or stronger strength, respectively, to turn on the PKMζ network. As expected, EPSC amplitude reached a value of only either 1 or 2 (Figure 3B), and the areas for EPSC_t = ∞ _= 1 and EPSC_t = ∞ _= 2 were identical to the areas for [PKMζ]_t = ∞ _= 0 and [PKMζ]_t = ∞ _= 0.72, respectively. This switch-like behavior indicated high nonlinearity of the system.

Up to this point, we have shown that the PKMζ network responds to stimuli in an all-or-nothing manner (Figures 2 and 3). Next, we performed a bifurcation analysis [32-34] to further illuminate the dynamics of the model and evaluate its parameter dependence. The model has seven parameters, including the rate parameters *j_1_*, *j_2_*, *j_3_*, and *j_4 _*and three time constants (see Methods), but we only needed to analyze the model with respect to the four rate parameters because the time constants do not affect the model equilibria (see additional file 1). *j_1 _*denotes the PKMζ synthesis rate relative to its decay rate, *j_2 _*and *j_3 _*denote the PKMζ-independent and PKMζ-dependent actin polymerization rates relative to the actin depolymerization rate, respectively, and *j_4 _*denotes the rate at which PKMζ mRNA is incorporated into the translational machinery relative to the rate of mRNA detachment from the machinery.

**Figure 4:**
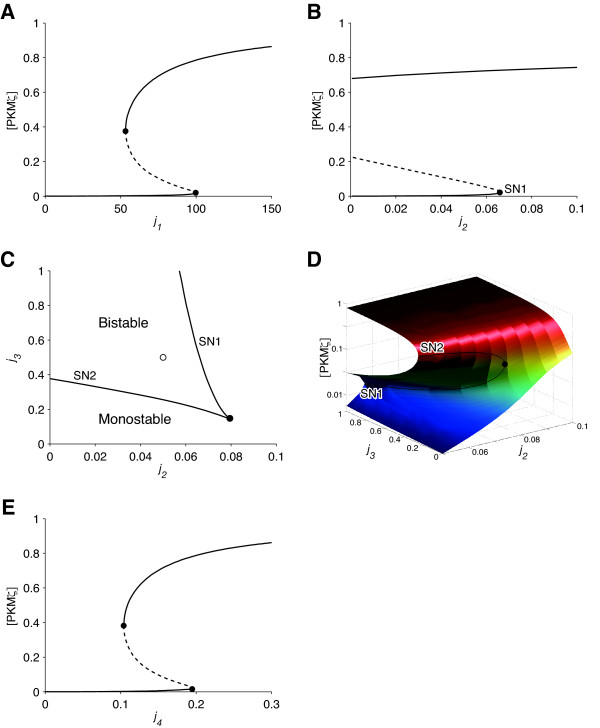
**Bifurcation analyses**. A) Bifurcation diagram with respect to the PKMζ synthesis rate, *j_1_*. B) Bifurcation analysis with respect to the PKMζ-independent actin polymerization rate, *j_2_*. Only one (SN1) of the two saddle node bifurcations is seen in this plot. C, D) Two-parameter bifurcation analysis with respect to *j_2_*, and the PKMζ-dependent actin polymerization rate, *j_3_*. C) A two-dimensional plot with *j_2 _*in the X-axis and *j_3 _*in the Y-axis. D) A three-dimensional plot with *j_2 _*in the X-axis, *j_3 _*in the Y-axis, and steady-state [PKMζ] in the Z-axis (log scale). E) Bifurcation analysis with respect to *j_4_*, the rate at which PKMζ mRNA is incorporated into the translational machinery. In panels A, B, and E, the solid and dashed lines indicate [PKMζ] at stable and unstable steady states, respectively; the circles denote saddle-node bifurcations. In panels C and D, the solid lines, solid circles, and open circle (only in panel C) indicate saddle-node bifurcations (SN1 and SN2), the cusp bifurcation point, and the default values of *j_1 _*and *j_2_*, respectively.

First, we varied the PKMζ synthesis rate *j_1 _*and tracked the equilibrium points of the model (Figure 4A). The horizontal and vertical axes show *j_1 _*and steady-state [PKMζ], respectively. The two solid lines denote stable steady states: the UP state (upper) and the DOWN state (lower). There were two saddle-node bifurcations (circles) at *j_1 _*= 53 and *j_1 _*= 100. When *j_1 _*< 53, only the DOWN state was stable; when *j_1 _*> 100, only the UP state was stable. When 53 ≤ *j_1 _*≤ 100, the system was bistable at the two stable steady states, the UP and DOWN states, which were separated by an unstable steady state (dashed line). The default value of *j_1 _*was 80 and within the range of bistability.

Next, we performed a bifurcation analysis with respect to the PKMζ-independent actin polymerization rate *j_2 _*and the PKMζ-dependent polymerization rate *j_3_*. Figure 4B shows *j_2 _*on the horizontal axis and steady-state [PKMζ] on the vertical axis. There was a saddle-node bifurcation (SN1) at *j_2 _*= 0.066, and the system was bistable when *j_2 _*< 0.066 and monostable at the UP state when *j_2 _*≥ 0.066. To further characterize the actin polymerization rates-dependent dynamics of the model, we performed two-parameter bifurcation analysis with respect to *j_2 _*and *j_3 _*(Figures 4C and 4D). The analysis revealed that two branches of the saddle node bifurcation curve (solid lines, SN1 and SN2) met tangentially at a point (solid circle). This type of bifurcation is mathematically termed the "cusp bifurcation" [34]. The parameter regions of monostability and bistability are indicated in Figure 4C. Loss of bistability at small values of *j_2 _*and *j_3 _*indicates that the F-actin-dependent facilitation of protein synthesis is essential for the bistability of the PKMζ network.

Lastly, we performed a bifurcation analysis in terms of *j_4_*, the rate at which PKMζ mRNA is incorporated into the translational machinery (Figure 4E). The system was monostable at the DOWN state when *j_4 _*< 0.10 and at the UP state when j_4 _> 0.19. It was bistable when 0.10 ≤ *j_4 _*≤ 0.19. The PKMζ network was shown to be bistable over wide ranges of the parameters *j_1_*, *j_2_*, *j_3_*, and *j_4_*, indicating its suitability as an engram.

### Comparison with previous experiments

Next, we simulated a variety of previous experiments to examine whether the model was able to explain their results.

#### PKMζ inhibition

**Figure 5:**
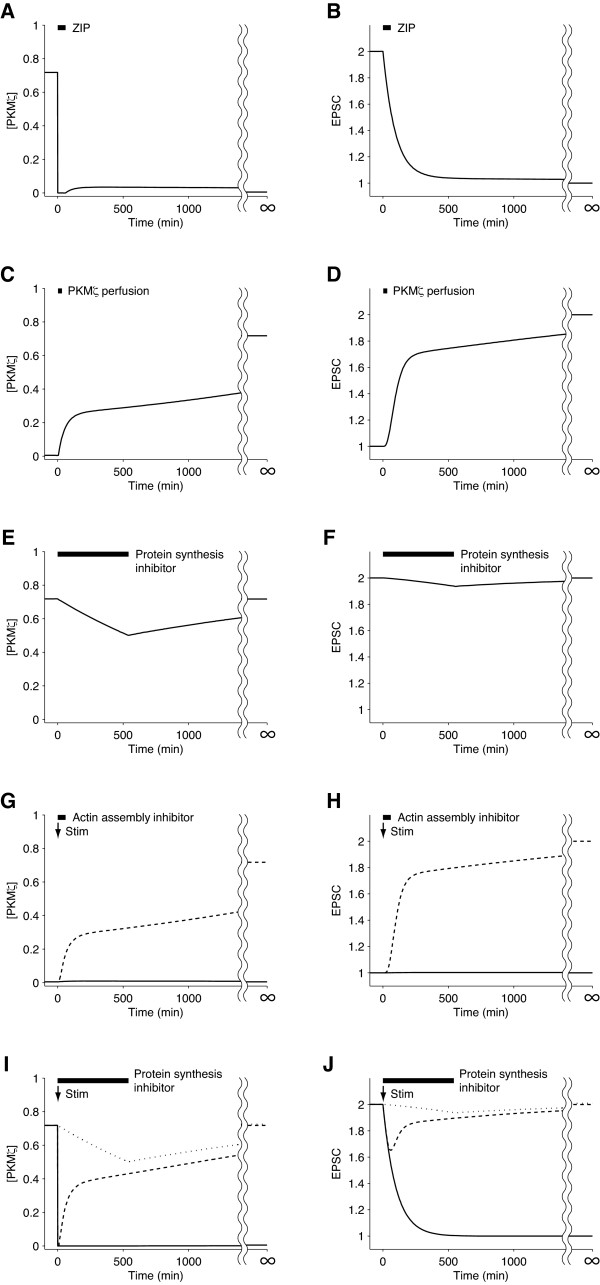
**Simulations reproducing previous experimental results**. Time courses of [PKMζ] (panels A, C, E, G, I) and EPSC amplitude (panels B, D, F, H, J) are shown. A, B) ZIP administration to a potentiated synapse. C, D) PKMζ perfusion. E, F) Protein synthesis inhibitor administration to a potentiated synapse. G, H) Stimulation in the presence (solid line) or absence (dashed line) of an actin assembly inhibitor. I, J) Reactivation in the presence (solid line) or absence (dashed line) of protein synthesis inhibitor. The dotted line corresponds to protein synthesis inhibition without reactivation. Arrows and bold bars indicate the onset of an LTP-inducing stimulus, Stim(t) = 25 (0 ≤ t < 30), and the duration of drug administration, respectively.

ZIP reverses LTP in hippocampal neurons even when applied five hours after LTP-inducing stimuli [9], indicating the essential role of PKMζ in L-LTP. We simulated this experiment by starting from the UP state and temporarily clamping [PKMζ] at zero for 60 minutes. The simulated time courses of [PKMζ] and EPSC amplitude are plotted in Figures 5A and 5B. When PKMζ was eliminated, EPSC amplitude began to gradually decrease. When [PKMζ] was unclamped at t = 60, it increased slightly, hit a peak, and decreased again, whereas EPSC amplitude constantly approached the DOWN state. Thus, the simulation result is consistent with the experimental result [9].

#### PKMζ introduction

The introduction of exogenous PKMζ has been shown to be sufficient to induce LTP in CA1 pyramidal neurons [25]. We simulated this finding by fixing [PKMζ] at 10 for 5 minutes to mimic exogenous PKMζ. The simulated time courses of endogenous [PKMζ] and EPSC amplitude are shown in Figures 5C and 5D. Similar to the experimental result, transient application of exogenous PKMζ activated PKMζ production and turned on the positive feedback loop, which perpetually maintained endogenous PKMζ expression and enhanced EPSC amplitude.

#### Protein synthesis inhibition

Transient inhibition of protein synthesis does not erode the consolidated memory of a behaving animal unless the memory is recalled simultaneously with the inhibition [35-37]. We simulated protein synthesis inhibition in a potentiated synapse by setting *j_1 _*to 0 during 0 ≤ t < 540. The duration of inhibition was based on a report that microinfusion of anisomycin into the hippocampus inhibited protein synthesis for 9 hours [38]. The simulated time courses of [PKMζ] and EPSC amplitude are plotted in Figures 5E and 5F. PKMζ was degraded so slowly that only a small portion was lost while *j_1 _*= 0 for 540 minutes, and the level returned to that of the UP state value when *j_1 _*was recovered. This result is consistent with the fact that transient protein synthesis inhibition does not usually affect consolidated memory [35-37].

#### Actin assembly inhibition

Actin assembly inhibitors such as cytochalasins and latrunculins inhibit LTP induction [39]. We simulated the temporal application of an actin assembly inhibitor by setting *j_2 _*and *j_3 _*to 0 for 60 minutes. The simulated time courses are plotted in Figures 5G and 5H. In agreement with experimental results, a stimulus that would have activated the PKMζ positive feedback loop in the control condition failed to do so when actin assembly was inhibited temporarily.

#### Reconsolidation

Upon retrieval, well-consolidated memories become labile and vulnerable to protein synthesis inhibitors (reactivation) before they are reconsolidated [35-37]. Reactivation is thought to trigger a consolidation-like process because reactivated memory and newly acquired memory have similar time courses of susceptibility to protein synthesis inhibition: they are intact in the short term but impaired in the long term when a protein synthesis inhibitor is administered either upon retrieval or upon *de novo *learning [40]. A reconsolidation-like process is also observable in slice electrophysiology [41]; synapses that are potentiated by tetanus stimulation are depotentiated when stimulated again in the presence of a protein synthesis inhibitor.

These findings indicate that carrier proteins of memory traces are depleted on retrieval and replaced by newly synthesized proteins to restore memory, as suggested previously [42]. Because PKMζ is a carrier of long-term memory, we, as well as other researchers, assumed an active mechanism that destroys PKMζ and induces its synthesis upon retrieval [1,13,37]. To examine whether this assumption is consistent with previous experimental results, we simulated the time courses of [PKMζ] and EPSC amplitude after reactivation (Figures 5I and 5J). Reactivation, which we supposed would destroy PKMζ protein, was mimicked by clamping [PKMζ] at zero during 0 ≤ t ≤ 10. *j_1 _*was set at 0 when 0 ≤ t < 540 and at the default value otherwise to imitate transient inhibition of protein synthesis. When the synapse was reactivated by a stimulus in the presence of a protein synthesis inhibitor, EPSC amplitude approached the DOWN state, and [PKMζ] did not recover after treatment (solid lines). By contrast, [PKMζ] and EPSC amplitude recovered and ended up in the UP state, when the synapse was treated with either a reactivating stimulus (dashed lines) or a protein synthesis inhibitor alone (dotted lines). These simulated results are in line with the process of reconsolidation seen in behaving animals and brain slices [35-37,41]. However, the consistency between the simulation results and experimental data does not necessarily prove our hypothesis that newly synthesized PKMζ replaces old PKMζ upon reactivation. Reconsolidation might also be explained by other plausible mechanisms.

## Discussion

Our model was extremely simple and lacked many of known pathways. Nevertheless, the model reproduced a variety of previous experimental results, suggesting that it captures the key characteristics of the PKMζ network. In this section, we go a step further and propose yet to be done experiments to examine the validity of the model.

### Reconsolidation

We assume that in the neuron, multiple nonlinear dynamics with various time constants are connected in tandem to store information stably and flexibly [13,18]. According to this model, memory reactivation switches off the slowest dynamic, the PKMζ network, and switches on the upstream dynamics (i.e., supralinear calcium increase and calcium-activated protein kinases) on the other hand. In Figures 5I and 5J, we demonstrated the consistency between the simulation results and previous experiments to underpin our hypothesis that upon memory reactivation, newly synthesized PKMζ replaces preexisting PKMζ. It is possible to take advantage of LTP reconsolidation *in vitro *(see Results section) [41] and verify this hypothesis. To examine whether PKMζ is degraded upon reactivation, hippocampal neurons are first treated with labeled amino acids, and then LTP is induced. Several hours later when LTP is consolidated, the neurons are either reactivated by another tetanus or left unstimulated (control) in the absence of labeled amino acids. According to our assumption of reactivation-triggered degradation, the amount of labeled PKMζ will rapidly decrease in reactivated neurons whereas it will remain constant in control neurons. To prove synthesis of PKMζ upon reactivation, LTP is first induced in hippocampal neurons in the absence of labeled amino acids. Several hours later, the neurons are either reactivated or left unstimulated (control) in the presence of labeled amino acids. Our hypothesis predicts that reactivated neurons will synthesize a greater amount of labeled PKMζ than control neurons, in which PKMζ is produced only to meet its turnover.

It might also be possible to examine our hypothesis *in vivo*; in inhibitory avoidance learning, animals are trained to associate the dark side of an experimental chamber with foot shocks, and this type of learning is known to induce LTP in the hippocampus [43]. First, the mouse PKMζ gene is replaced with a PKMζ-GFP chimeric gene. Then, the engineered mice are trained for an inhibitory avoidance task. Several days later, the preexisting PKMζ-GFP in the hippocampus is photobleached, and the mice are divided into three groups: the reactivation group, the reactivation-protein synthesis inhibition group, and the control group. The reactivation group mice are exposed to the experimental chamber to reactivate the fear memory. Those in the reactivation-protein synthesis inhibition group are first administered a protein synthesis inhibitor and then exposed to the experimental chamber. Control mice are exposed to another chamber distinct from the one used in the training sessions. It will be possible to quantify the newly synthesized PKMζ-GFP by using fluorescence microscopy and the total PKMζ-GFP (synthesized either before or after photobleaching) by an immunological method.

Based on our hypothesis, a series of predictions can be made. In the reactivation group, PKMζ-GFP fluorescence will increase, whereas the total amount of PKMζ-GFP will remain constant. In the reactivation-protein synthesis inhibition group, both the fluorescence and total amount of the chimeric protein will decrease. In the control group, the fluorescence will stay at a low level, and the total amount of PKMζ-GFP will remain constant.

### F-actin stabilizer

**Figure 6:**
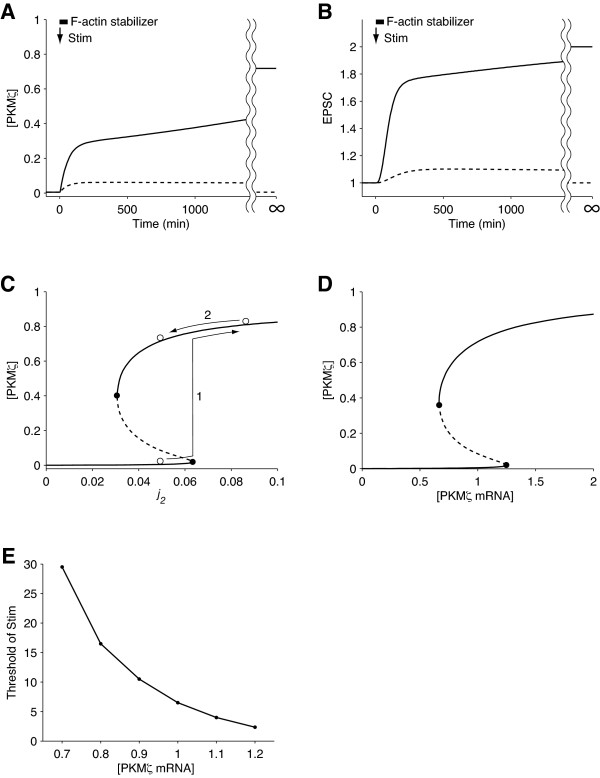
**Simulations predicting experimental results**. Time courses of A) [PKMζ] and B) EPSC amplitude after a weak stimulus, Stim(t) = 5 (0 ≤ t < 30), in the presence (solid line) or absence (dashed line) of a F-actin stabilizer. C) Steady-state [PKMζ] versus an actin polymerization rate, *j_2_*. The other actin polymerization rate, *j_3, _*was treated as a dependent parameter (*j_3 _*= 10*j_2_*). D, E) [PKMζ mRNA] and bistability. D) Steady-state [PKMζ] versus the bifurcation parameter, [PKMζ mRNA]. E) The threshold [PKMζ mRNA] to perpetually activate the system.

An F-actin stabilizer such as phalloidin [44] increases the actin polymerization rate relatively to the actin depolymerization rate. To predict the effect of a F-actin stabilizer on LTP, we omitted the decay term temporarily (0 ≤ t ≤ 60) from the ODE for F-actin (Equation 2) and simulated the time courses of [PKMζ] (Figure 6A) and EPSC amplitude (Figure 6B). In the presence of the F-actin stabilizer, the system resulted in the UP state when treated with a weak stimulus that would be insufficient to turn on the network in the control condition.

Next, we investigated how the F-actin stabilizer changes the dynamics of the model. A bifurcation diagram with respect to actin stability (Figure 6C) was obtained by slicing the two-parameter (*j_2 _*and *j_3_*) bifurcation plot (Figure 4D) with a perpendicular plane *j_3 _*= a*j_2_*, where "a" is a positive constant (a = 10 in Figure 6C). The horizontal and vertical axes of the slice show *j_2 _*and the steady state [PKMζ], respectively. Solid circles indicate saddle-node bifurcations. *j_2 _*divided the dynamics of the model into three phases. The system was bistable when *j_2 _*was between 0.031 and 0.063 and monostable when *j_2 _*was outside of this range. A small rightward shift of *j_2 _*from the default value did not change [PKMζ] significantly, but a large shift thrust the system beyond the right bifurcation point and brought it to the UP state (arrow 1). Subsequent withdrawal of the F-actin stabilizer did not restore the DOWN state (arrow 2) and showed hysteresis, a characteristic feature of bistable systems. These simulations predict that an F-actin stabilizer lowers the threshold of stimulation for LTP induction at low doses and induces LTP without stimuli at higher doses.

### Variation of mRNA concentration

In recent years, remarkable *in vivo *techniques for gene transfer and local gene knockdown in the brain have been developed [45,46]. In our model, we assumed a constant level of PKMζ mRNA, but such methods will enable variation of [PKMζ mRNA]. We predicted its outcome by extending the model and performing a bifurcation analysis with respect to [PKMζ mRNA] (default value of 1) as a bifurcation parameter (Figure 6D). The dynamics of the PKMζ network was dependent on [PKMζ mRNA] and had three distinct phases. The system was monostable at the DOWN state when [PKMζ mRNA] < 0.67 and at the UP state when [PKMζ mRNA] > 1.2. The system was bistable when 0.67 ≤ [PKMζ mRNA] ≤ 1.2.

As [PKMζ mRNA] increased, the saddle point approached the DOWN state (Figure 6D). One may assume that when the saddle point is closer to the DOWN state, the Stim threshold for switching on the system will be lower. Unfortunately, however, that assumption is not obvious from a two-dimensional projection (Figure 6D) of a system with many more dimensions. To obtain a clear view, we varied [PKMζ mRNA] and found the threshold of Stim (a square wave lasting for 30 minutes) necessary for turning the system on. Figure 6E plots the threshold value of Stim against [PKMζ mRNA]. As predicted, the concentration and input threshold were negatively correlated. It would be possible to verify this prediction *in vitro *and *in vivo *by introducing a PKMζ gene construct into the hippocampus. Moderate PKMζ overexpression will lower the threshold for LTP induction and alter learning efficacy by increasing the sensitivity of the PKMζ network to input, whereas an overdose of the gene will induce LTP without stimuli and hinder learning ability by destroying the bistability of the system. Silencing PKMζ expression by RNA interference will also destroy the bistability of the system and prevent LTP and learning.

### Bistable positive feedback loop models

Bistable networks are ubiquitous in biology. In particular, combination of positive feedback and nonlinearity is a very common mechanism for creating bistability. For instance, the MAPK positive feedback loop makes a bistable switch and plays crucial roles in development and memory [14,47,48]. Theoretical studies have demonstrated that nonlinearity of the pathway arises from dual phophorylations of the MAPK cascade; MAPK kinase kinase dually phosphorylates and activates MAPK kinase, and dually-phosphorylated MAPK kinase then dually phosphorylates MAPK [14,49]. CaMKII affords another example. According to a simulation study, the activity of CaMKII is bistable because its autophosphorylation provides positive feedback and the rate of autophosphorylation is nonlinearly dependent on the number of phosphorylated subunits [50]. In contrast to the MAPK and CaMKII models, our PKMζ model is unique since bistability arises from combination of positive feedback and pathway converge (Figure 4C). Aslam et al. modeled a bistable positive feedback loop consisting of CaMKII and a translational regulator, where CaMKII autophosphorylation activates translation of CaMKII [51]. Their model is similar to ours in that translation plays a crucial role in bistability.

### Cerebellar long-term depression

Long-term depression of the parallel fiber-Purkinje cell synapse is thought to be the cellular substrate of cerebellar learning [52]. Cerebellar LTD shows remarkable similarity to hippocampal LTP, although they have different directions of plasticity and involve different receptor subunits; both require large calcium transients and subsequent activation of CaMKII and PKC, and both follow AMPAR phosphorylation [52-56]. In the cerebellar Purkinje cell, stimuli induce a supralinear calcium influx, which activates MAPK and PKC in an all-or-none manner [48,57-60]. MAPK and PKC are engaged in cerebellar LTD only in the early phase (approximately 30 minutes) [48,60], and what maintains LTD in the later phase is not yet known. PKMζ, the long-term memory trace in a variety of brain regions [1,61], is also found in the cerebellar cortex [61], suggesting its potential involvement in cerebellar LTD [13,18,62]. This study mainly focuses on hippocampal L-LTP, but considering the similarity, it might also explain the mechanism for cerebellar memory.

## Conclusions

We have shown here that the PKMζ network is robustly bistable, supporting its pivotal role in long-term memory. Obviously, PKMζ is not the sole mechanism for long-term memory; expression of various proteins, morphological changes, and synaptogenesis are also very important [63-69]. Interaction of molecular pathways that have different time scales is also important for memory stability [70]. Further experimental and computational studies will be necessary to address how these processes interact and cooperate and which process is the most crucial in retaining memory.

## Methods

### Simulation of the biochemical reactions

Figure 1 illustrates the pathways of the PKMζ network model. Because qualitative data were not available for any of the pathways, we presumed the simplest case where each reaction was a first-order reaction. The following set of reactions describes the molecular interactions of the PKMζ network. PKMζ protein stimulates translation of its own mRNA (Figure 1 arrow 2) [25] and promotes actin polymerization (Figure 1 arrow 3) [26-29]. F-actin facilitates PKMζ-induced PKMζ synthesis (Figure 1 arrow 4) [31]. Stim(t) represents the collective activity of protein kinases, including PKC, MAPK, and CaMKII, that induce PKMζ expression (Figure 1 arrow 1) [2,7,12]; its basal value was 0.003, representing the background activity of the protein kinases.

The three ODEs that describe the reactions are as follows:

(1) $$\tau_{1}\frac{d[PKM\zeta ]}{dt}=j_{1}[RNA_{active}]\left(1-[PKM\zeta ]\right)-[PKM\zeta ]$$

(2) $$\tau_{2}\frac{d[FActin]}{dt}=\left(j_{2}+j_{3}[PKM\zeta ]\right)\left(1-[FActin]\right)-[FActin]$$

(3) $$\tau_{3}\frac{d[RNA_{active}]}{dt}=j_{4}[FActin]\left([PKM\zeta ]+Stim\left(t\right)\right)\left(1-[RNA_{active}]\right)-[RNA_{active}]$$

, where [PKMζ], [FActin], and [RNA_active_] denote the concentrations of PKMζ protein, actin protein molecules assembled into F-actin, and PKMζ mRNA recruited into the local translational machinery ('active'), respectively.

In the model, concentrations are unitless quantities. In the future, when quantitative data are available, it would be simple to convert them into quantities with units (such as micromolar). Concentrations of total actin, including F-actin and G-actin (actin monomer), and total PKMζ mRNA (mRNA in and out of the translational machinery) are both assumed to be constant and designated values of one since neither actin or PKMζ mRNA has been shown to increase or decrease upon LTP induction. Assuming biological regulation and resource limitation, the upper limit of [PKMζ] was set to one. The system was still bistable without this constraint (data not shown). Time has a unit of minutes. The time constants *τ_1_*, *τ_2_*, and *τ_3 _*were determined so that PKMζ turnover, actin polymerization, and protein synthesis would take place in realistic time scales. The parameters *j_1_*, *j_2_*, *j_3_*, and *j_4 _*denote reaction rates relative to the exponential decay of each variable: *j_1 _*designates the PKMζ synthesis rate, *j_2 _*and *j_3 _*denote the PKMζ-independent and PKMζ-dependent actin polymerization rates, respectively, and *j_4 _*is the rate at which PKMζ mRNA is incorporated into the translational machinery.

Equation 1 describes the processes in which PKMζ is translated from its active mRNA and degraded (Figure 1 arrow 1). The production rate has a time scale of 10 minutes (depending on [RNA_active_]), which is comparable to experimental results [2]. Protein degradation was assumed to have a time scale of one day because the catalytic domain of PKCζ, whose amino acid sequence is identical to that of full-length PKMζ, has a half-life of at least one day [71]. Equation 2 refers to the reactions in which G-actin is transformed to F-actin and vice versa. The forward step is at least partly dependent on PKCζ activity (Figure 1 arrow 3) [26-29]. Actin turnover in the dendritic spine was assumed to have a time scale of tens of seconds, based on previous experiments [72,73]. Equation 3 describes the process in which PKMζ mRNA is recruited into translation machinery in a [PKMζ]- and [FActin]-dependent manner (Figure 1 arrows 2, 3) [25].

PKMζ enhances EPSC by stabilizing AMPA receptors in postsynaptic sites through an NSF/GluR2-dependent pathway [3-5]. AMPA receptors are composed of four subunits, including two GluR2 s and two others [74,75], and NSF is thought to interact with each GluR2 subunit [76]. Therefore, we presumed that PKMζ-dependent EPSC changes were a second-order reaction. EPSC was estimated by solving the following ODE:

(4) $$\tau_{4}\frac{d\text{EPSC}}{dt}=j_{5}\left({\text{EPSC}}_{\text{UP}}-\text{EPSC}\right)\frac{{[\text{PKM}\zeta \text{]}}^{\text{2}}}{{[\text{PKM}\zeta \text{]}}_{\text{UP}}^{\text{2}}}-\text{EPSC}+j_{6}$$

EPSC has an amplitude of 1 (arbitrary unit) in the DOWN state and an amplitude of 2 in the UP state (EPSC_UP_). The decay term is based on the findings that without PKMζ activity, EPSC amplitude in potentiated neurons approaches its baseline level [9,25]. The time constant *τ_4 _*was derived from experiments [9,25]. *j_5 _*was set to meet the initial rate of EPSC increase after postsynaptic introduction of exogenous PKMζ [9,25]. *j_6 _*determines the basal level of EPSC.

Default parameters were represented by the following values: *τ_1 _*= 1500, *τ_2 _*= 0.5, *τ_3 _*= 60, *τ_4 _*= 100, *j_1 _*= 80, *j_2 _*= 0.05, *j_3 _*= 0.5, *j_4 _*= 0.16, *j_5 _*= 14, and *j_6 _*= 0.89. We implemented the model equations into the software package XPPAUT [32] and performed numerical integrations and bifurcation analyses.

## Authors' contributions

HO designed and performed the study and drafted the manuscript. MK participated in the design the study and helped to draft the manuscript. Both authors read and approved the final manuscript.

## Supplementary Material

Additional file 1 **Time constants do not affect model equilibria**. This text explains that the dynamics of the model is independent of its time constants. Click here for file
