# Supplementary material for: The protein kinase Mζ network as a bistable switch to store neuronal memory
Source: BMC Syst Biol. 2010 Dec 31;4:181. doi: 10.1186/1752-0509-4-181 (PMC3022653; doi:10.1186/1752-0509-4-181)
Supplement: Additional file 1 — Time constants do not affect model equilibria. This text explains that the dynamics of the model is independent of its time constants. [file 1752-0509-4-181-S1.PDF]

Additonal file 1. Time constants do not affect model equilibria.

The Jacobian matrix of the model can be described as $\boldsymbol{J} = diag(\tau_1^{-1}, \tau_2^{-1}, \tau_3^{-1})\boldsymbol{A}$, where $\boldsymbol{A}$ is a 3-by-3 matrix, each row of which designates partial derivatives of the right-hand side of the corresponding ODE evaluated at the equilibrium point with respect to state variables. Bifurcation theory states that the Jacobian matrix has a simple zero eigenvalue at a saddle-node bifurcation point [1-3]. That means that the characteristic polynomial of $\boldsymbol{J}$ has a zero degree term (i.e., det$\boldsymbol{J}$) of zero, and therefore, $\frac{detA}{\tau_1\tau_2\tau_3} = 0$. This equation indicates that the saddle-node bifurcation of the model is independent of the time constants because the equality holds irrespective of them. Thus, the bifurcation can be thoroughly analyzed in a relatively low-dimensional parameter space consisting of j_1, j_2, j_3, and j_4. In contrast to the saddle-node bifurcation of the model, its Hopf bifurcation is dependent on the time constants, which means that the higher-dimensional parameter space needs to be explored, making analysis much more difficult. However, despite an extensive search, the model did not exhibit any Hopf bifurcation around the default values of the parameters.

References

1. Ermentrout B: *Simulating, Analyzing, and Animating Dynamical Systems: A Guide to XPPAUT for Researchers and Students.* 1st edn:

Society for Industrial Mathematics; 2002.

2. Fall CP, Marland ES, Wagner JM, Tyson JJ: *Computational Cell Biology.* New York: Springer-Verlag; 2002.

3. Kuznetsov YA: *Elements of applied bifurcation theory.* 3rd edn. New York: Springer; 2004.
